# The Association between Diabetes Mellitus and Nonarteritic Anterior Ischemic Optic Neuropathy: A Systematic Review and Meta-Analysis

**DOI:** 10.1371/journal.pone.0076653

**Published:** 2013-09-30

**Authors:** Ting Chen, Delu Song, Guangliang Shan, Ke Wang, Yiwei Wang, Jin Ma, Yong Zhong

**Affiliations:** 1 Department of Ophthalmology, Peking Union Medical College Hospital, Chinese Academy of Medical Sciences and Peking Union Medical College, Beijing, China; 2 Scheie Eye Institute, University of Pennsylvania Perelman School of Medicine, Philadelphia, Pennsylvania, United States of America; 3 Department of Epidemiology and Statistics, Institute of Basic Medical Science, Chinese Academy of Medical Sciences and Peking Union Medical College, Beijing, China; Universidad Peruana Cayetano Heredia, Peru

## Abstract

**Background:**

The association of diabetes mellitus (DM) with nonarteritic anterior ischemic optic neuropathy (NAION) has been inconclusive.

**Purpose:**

To determine whether DM is associated with an increased risk of NAION.

**Methods:**

A comprehensive literature search was performed for published studies reporting both DM and NAION based on PubMed and EMBASE. After reviewing characteristics of all the included studies systematically, meta-analytical method was employed to calculate the pooled odds ratio (OR) and associated 95% confidence interval (CI) from random-effects models. Heterogeneity was assessed by Q-statistic test. Funnel Plot, Begg's and Egger's linear regression test were applied to evaluate publication bias. A sensitivity analysis and meta-regression analysis were also performed to assess the robustness of results.

**Results:**

2,096 participants from 12 case-control studies were pooled for a meta-analysis. The result of meta-analysis of these studies indicated that DM is associated with increased risk of NAION (pooled OR = 1.64, 95% CI = 1.17–2.30; *P* = 0.004). Sensitivity analysis indicated our findings are robust, and meta-regression analysis revealed no significant effect in terms of geographical area, gender, age of patients with NAION, the year of the publication, source of the controls, and sample size (all p>0.05). Evidence of publication bias was not observed in our study.

**Conclusion:**

Meta-analysis suggests that DM might be associated with increased risk of NAION.

## Introduction

Nonarteritic anterior ischemic optic neuropathy (NAION) is the most common acute optic neuropathy in people aged 50 years and older, with an estimated annual incidence of 2.3 to 10.3 per 100,000 persons in the United States [1], [2]. It usually presents with sudden painless unilateral visual loss, typical visual field defects, relative afferent papillary defect and characteristic fundus changes (swollen, hyperemic discs or presence of peripapillary hemorrhages) [3].

Although the precise cause of NAION remains elusive, the etiology of NAION is believed to be multifactorial. The proposed risk factors include: (1) the anatomic predisposition of a “crowded” optic disk [4], [5]; (2) systematic conditions, such as hypertension [6], [7], diabetes mellitus (DM) [8]–[10], hyperlipidemia [6], [10], [11], sleep apnea syndrome[12], [13] and nocturnal hypotension [14], [15]; (3) side effects of some medical or surgical interventions, such as erectile dysfunction drugs [16], [17], amiodarone [18], [19], instance liposuction and hemodialysis [20]; and (4) genetic factors [20]–[23]. Among these factors, DM, an important risk factor, needs further investigation.

The association between DM and NAION, however, is controversial. Although DM was found to significantly increase the risk of NAION in some publications [8], [10], [11], [13], [24], others found only borderline relationship [25], [26] or even no association between these two entities [6], [20], [27]–[31]. To address this issue, a comprehensive evaluation using meta-analysis of published case-control studies based on a thorough systematic literature review was carried out. Our analysis showed that DM might be associated with increased risk of NAION. Since the number of DM patients is increasing world-widely due to urbanization, population growth and aging, and the prevalence of diabetes for all age-groups worldwide was estimated to reach 4.4% in 2030 by the World Health Organization [32], the results of our study might have profound clinical and public health significance.

## Materials and Methods

### Search Strategy

We searched the electronic databases of PubMed and EMBASE (up to October 2012) for relevant papers using the following terms: “nonarteritic anterior ischemic optic neuropathy”, “non-arteritic anterior ischemic optic neuropathy”, “risk factor”, AND “diabetes mellitus”, limiting the search to English-language articles. The reference lists of the retrieved articles were also reviewed to identify publications on the same topic. No protocol exists for this systematic review.

### Study Selection

To be qualified for this meta-analysis, studies had to meet the following criteria: (1) the studies had to be unrelated case-control study and the control group be matched with the NAION group for age and gender, representing an assessment of the association between the DM and NAION; (2) the study must present sufficient dichotomous data on DM and NAION; (3) the study must recruit diagnosed NAION patients and the outcome had to be NAION related. Studies were excluded if one of the followings met: (1) the design was based on family or sibling pairs; (2) the full text of the studies can't be retrieved; (3) the type of the study was the review, case related, comment/letter/note, or conference related; (4) the purpose of the study was exclusively to describe the characteristic of NAION; (5) the study focus on therapeutics of NAION rather than risk factors. For multiple publications from the same study group, the largest dataset and recent results were chosen.

### Data Extraction

A standardized reporting form was used to abstract the data from each study: first author's name, year of the publication, country in which the study was conducted, sample size, source of the controls, mean age, gender ratio of all participants. Data were extracted independently by two investigators (TC, JM). The results were compared and disagreements were resolved by consensus.

### Quality Assessment

Two investigators (TC, JM) performed the quality assessment by using the Newcastle-Ottawa scale for included studies [33]. This scale allocates a maximum of eight stars for the best quality of selection, comparability and exposure. The four criteria in evaluating our selection are: (1) is the NAION definition adequate; (2) representativeness of the NAION; (3) selection of controls; (4) definition of controls. Four stars can be allotted. The comparability is about the comparability of NAION and controls on the bias of the design or analysis and a maximum of two stars can be allotted. The exposure referring to DM in our analysis has two aspects: (1) ascertainment of DM; (2) non-response rate and can be allotted two stars. The two authors discussed the implementation of this assessment tool and agreed on a method of implementation before their independent assessments of the studies.

### Statistical Analyses

Pooled odd ratio (OR) and its 95% confidence interval (CI) were used to assess the association between DM and NAION. Heterogeneity between the studies was evaluated with Q statistic-test and *I* ^2^ statistic [34]. If *P* value from Q statistic-test is less than 0.10, the between-study heterogeneity was considered to be significant. *I* ^2^ statistic ranges from 0% and 100%, with 0% representing no heterogeneity and larger values representing larger heterogeneity (*I* ^2^ = 0–25% indicates no or mild heterogeneity; *I* ^2^ = 25–50% for moderate heterogeneity; *I* ^2^ = 50–75% for large heterogeneity; and *I* ^2^ = 75–100% for extreme heterogeneity) [35]. When inter-studies heterogeneity based on Q statistic-test and *I* ^2^ statistic was absent, the fixed-effects model was used to calculate the pooled OR. Otherwise, a random-effects model was used. The meta-analysis results were summarized graphically using a Forest Plot.

Publication bias was investigated by Funnel Plot. Funnel Plot asymmetry was assessed by using the method of Begg's and Egger's linear regression test [36]. A sensitivity analysis was performed by excluding one study at a time to indentify the potential influence of the individual data set on the pooled OR. Univariate meta-regression analysis was used to explore the effect of study characteristics on the estimate of association. The meta-analysis was performed using Stata software (version 11.0; Stata Corporation, College Station, TX). Two-sided *P*<0.05 was considered statistically significant (except for tests of heterogeneity where a level of 0.10 was used).

## Results

### Study Characteristics

**Figure 1:**
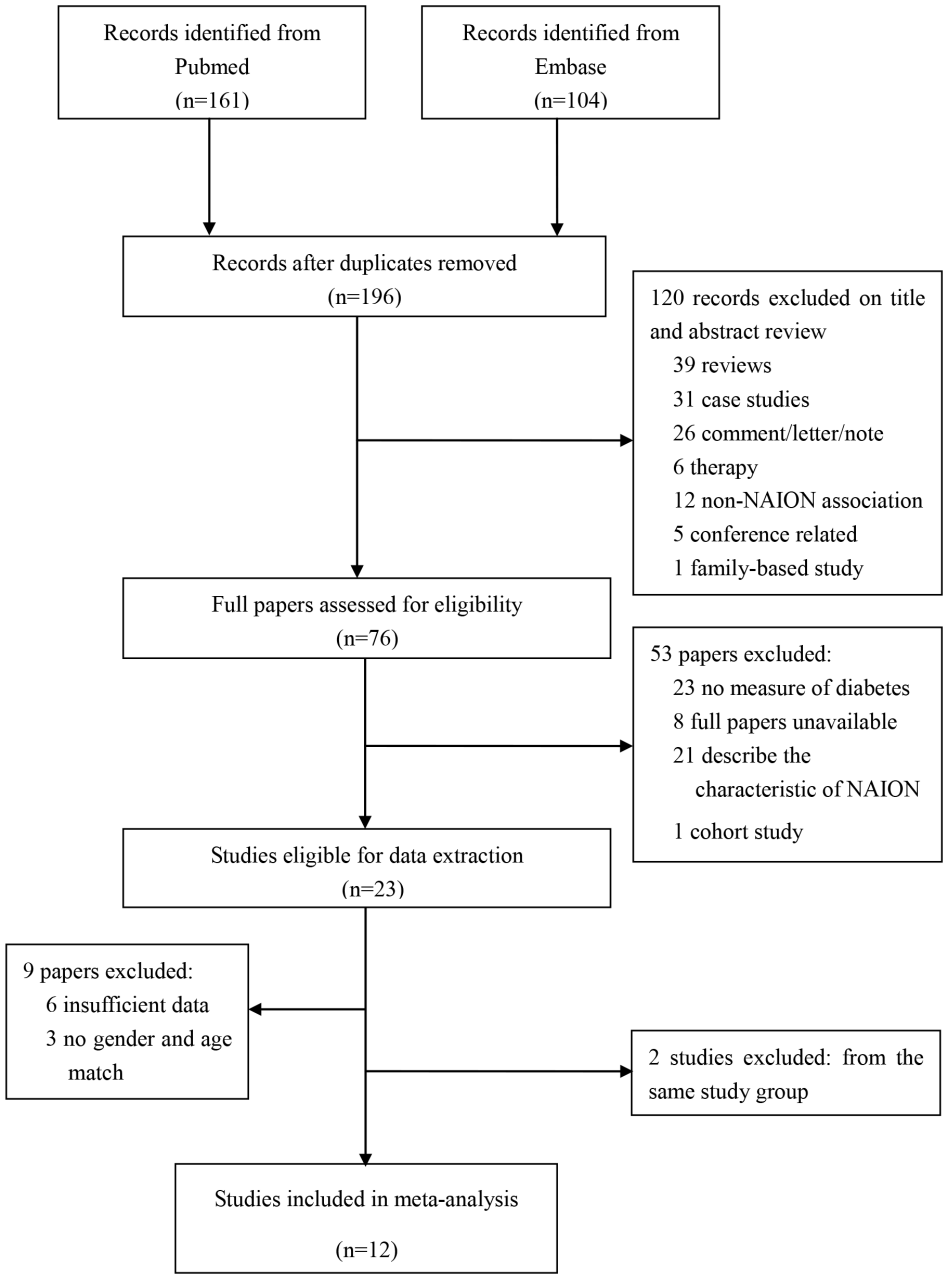
Flow diagram outlining the selection process for studies in the systematic review and meta-analysis.

We identified 265 articles from the database in total, with 161 from Pubmed and 104 from Embase. After removal of 69 duplicate articles, there were 196 articles (Figure 1) left. According to the exclusion criteria, 120 records were excluded after reviewing of their titles and abstracts and 53 papers were excluded after reading the full-texted papers, and 23 papers were remained for data extraction. Because of insufficient data and no gender- and age-matched controls, 9 papers were excluded as well. Finally, 14 articles met our inclusion criteria. The articles published by Li et al [13] and McGwin et al [37] were originated from the same study, two articles by Weger et al [24], [38] were also from the same study, therefore the most recent articles with larger dataset [13], [24] were used in our analysis. One study [25] included 2 independent sub-studies, in which the controls were chosen from different population. The data of controls were treated separately. After qualification, 12 studies were included in the meta-analysis. Characteristics of these studies are presented in Table 1. In these studies, 4 were conducted in the United States, 6 in Europe (Greece, Italy, Austria and United Kingdom) and 2 in Israel. A total of 2,096 participants were included in these 12 case-control studies with sample size ranging from 82 to 420. The mean value of all the selection, comparability and exposure for the included studies was 5.0 stars (Table 2).

**Table 1:** Main characteristics of the case-control studies included in the meta-analysis, 1991–2011.

First Author, Year (Reference No.)	Country of study location	Total sample size	Age, Years NAION	Age, Years Control	Male, (%) NAION	Male, %Control	Source of controls
Markoula, S 2011 [21]	Greece	123	66.2	65.6	29 (61.7)	47 (61.8)	Healthy volunteers
Felekis, T 2010 [20]	Greece	137	63.4	66.3	50 (64.9)	32 (53.3)	Cataract operation
Giambene,B 2009 [6]	Italy	255	65	65	39(45.9)	75(44.1)	Relatives or friends of patients
Kesler, A 2009 [28]	Israel	184	62.5	61.9	20(60.6)	91(60.3)	Routine health examination
Pinna, A 2008 [39]	Italy	420	63.6	_	68(48.6)	136(48.6)	Cataract register
Li, J 2007 [13]	USA	146	63.1	63.5	38(52.1)	38(52.1)	Ophthalmology clinic, non-NAION
Deramo, VA 2003 [11]	USA	111	43.2	43.0	25(68)	50(68)	Internal medicine practice
Weger, M 2002 [24]	Austria	142	68.1	68.3	41(57.7)	41(57.7)	Hospital-based, non-NAION
Salomon, O 1999 [10]	Israel	151	62	66	45(74)	53(59)	Eye Institute, non-NAION
Jacobson,DM a 1997 [25]	USA	204	68	–	30(59)	90(59)	MESA
Jacobson,DMb 1997 [25]	USA	102	68	–	30(59)	30(59)	Routine comprehensive medical examination
Talks, S J 1995 [30]	UK	82	66.7	66.9	27(65.9)	27(65.9)	Pre-operative cataract assessment clinic
Kalenak, JW 1991 [31]	USA	90	66.7	66.6	21(46.7)	21(46.7)	Normal eye examination

**Table 2:** Assessment of study quality.

First Author, Year	Selection	Comparability	Exposure
Markoula, S, 2011	★★★★	★	★
Felekis, T, 2010	★★★	★	★
Giambene,B, 2009	★★	★	★
Kesler, A, 2009	★★	★	★★
Pinna, A, 2008	★★	★	★
Li, J, 2007	★★	★	★★
Deramo, VA, 2003	★★	★	★
Weger, M, 2002	★★★	★	★
Salomon, O, 1999	★★★	★	★
Jacobson,DM a, 1997	★★	★	★★
Jacobson,DM a, 1997	★★★	★	★★
Talks, S J, 1995	★★	★★	★★
Kalenak, JW, 1991	★★	★	★★

### Pooled Estimates of the Association between DM and NAION

**Figure 2:**
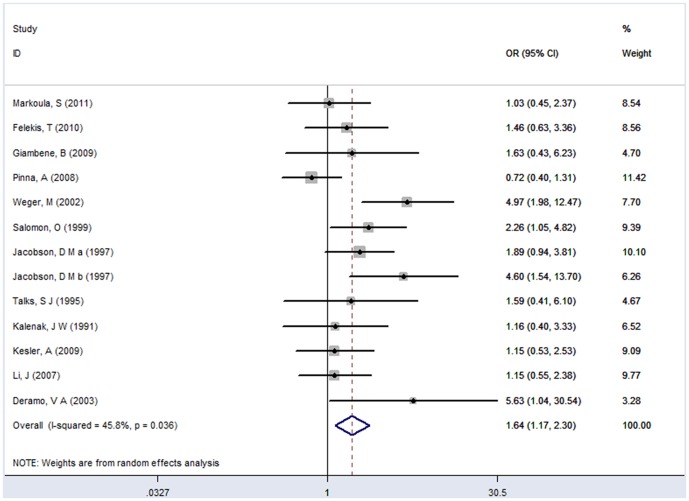
Random effects meta-analysis investigating the association of DM with NAION. CI =  confidence interval; OR =  odds ratio; DM =  diabetes mellitus; NAION =  nonarteritic anterior ischemic optic neuropathy.

The summary risk estimates for DM and NAION were plotted in Figure 2. Individuals with DM had a significantly increased risk of NAION, compared with non-diabetic individuals (pooled OR = 1.64, 95% CI = 1.17–2.30; random-effects *P* = 0.004). The Q-statistic test and *I* ^2^ statistic indicated a moderate but significant between-study heterogeneity across the included studies (Q = 22.14, df = 12, *P* = 0.036, *I* ^2^ = 45.8%).

### Sensitivity Analysis

Sensitivity analysis (Table 3) showed that the pooled OR estimates were statistically significant (i.e. all 95% CIs did not include 1) no matter what study was excluded from analysis, suggesting the robustness of results. This analysis also revealed that 2 studies, by Pinna et al [39] and Weger et al [24], were the main origins of heterogeneity. The I^2^ measure for DM significantly declined from 45.8% to 26.7% (Q = 15.01, df = 11, P = 0.18) after removing the study by Pinna et al [39] and to 28.3% (Q = 15.34, df = 11, P = 0.17) after removing the study by Weger et al [24]. Homogeneity was achieved after excluding these 2 studies together (Q = 9.67, df = 10, *P* = 0.47, *I* ^2^ = 0.0%), an OR of 1.62 was obtained (95% CI, 1.24–2.13, fixed-effects, *P* = 0.00).

**Table 3:** Results of leave-one-out sensitivity analysis.

	Pooled OR (95% CI)		Heterogeneity*	
Study Excluded	Fixed-Effects Model	Random-Effects Model	*I* ^2^ (%)	p Value
Markoula, S,2011	1.60 (1.26–2.04)	1.72 (1.20–2.47)	48.1	0.031
Felekis, T,2010	1.56 (1.22–1.98)	1.67 (1.15–2.42)	50.3	0.023
Giambene, B,2009	1.55 (1.22–1.96)	1.65 (1.15–2.36)	50.3	0.023
Kesler, A,2009	1.59(1.25–2.03)	1.71 (1.18–2.47)	49.1	0.028
Pinna, A,2008	1.80 (1.39–2.33)	1.80 (1.31–2.46)	26.7	0.182
Li, J,2007	1.60 (1.25–2.05)	1.71 (1.18–2.49)	48.8	0.029
Deramo, VA,2003	1.51 (1.19–1.90)	1.57 (1.12–2.19)	44.5	0.048
Weger, M,2002	1.40 (1.10–1.79)	1.46 (1.08–1.97)	28.3	0.168
Salomon, O,1999	1.49 (1.17–1.90)	1.59 (1.11–2.30)	47.7	0.033
Jacobson, DM a,1997	1.51 (1.18–1.93)	1.63(1.12–2.37)	49.4	0.026
Jacobson, DM b,1997	1.46 (1.15–1.85)	1.52 (1.09–2.10)	38.9	0.081
Talks, S J,1995	1.55 (1.22–1.96)	1.65(1.15–2.36)	50.3	0.023
Kalenak, J W,1991	1.57 (1.24–1.99)	1.69 (1.18–2.43)	49.7	0.025

CI =  confidence interval; OR =  odds ratio.

* Heterogeneity between the studies was evaluated with Q statistic-test and was considered significant if P<0.05.

### Meta-Regression

The univariate meta-regression analysis showed no statistically significant effect of 6 variables on the association, including sample size (*P* = 0.07), percentage of male (*P* = 0.11), mean age of patients with NAION (*P* = 0.72), the year of the publication (*P* = 0.13), source of the controls (*P* = 0.73), geographical area (*P* = 0.62).

### Publication Bias

**Figure 3:**
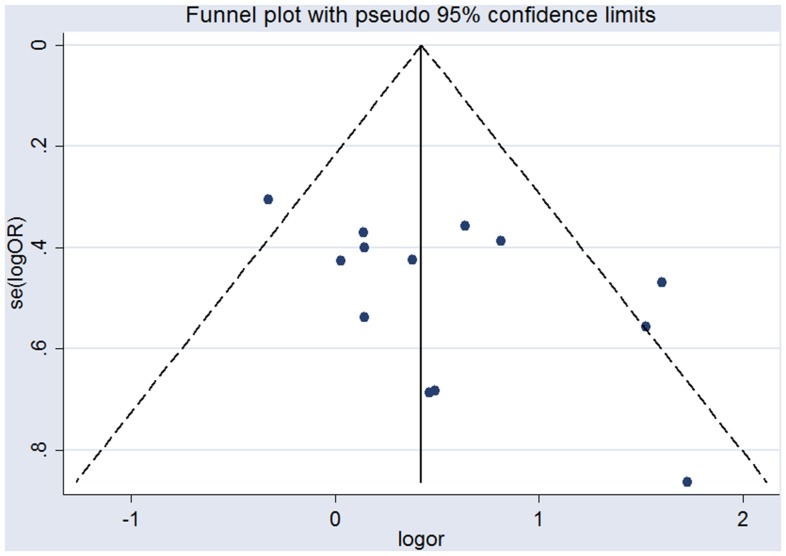
Funnel plot of all studies evaluating the association between DM and NAION. DM =  diabetes mellitus; NAION =  nonarteritic anterior ischemic optic neuropathy.

Funnel Plot and Begg's and Egger's test were conducted to assess the publication bias of the enclosed literature. As seen in Figure 3, the funnel plot appears symmetric, indicating absence of substantial publication bias. Evidence of publication bias was also not seen with the Egger or Begg tests (Egger *P* = 0.07 and Begg *P* = 0.13).

## Discussion

To the best of our knowledge, this is the first meta-analysis investigating the association between DM and NAION. The results of our meta-analysis showed that relative to non-diabetes controls, individuals with DM have increased risk of NAION, with a pooled OR of 1.64 (*P* = 0.004). Sensitivity analysis indicated that our statistical results are robust and are unlikely to be due to publication bias.

Due to the high incidence and prevalence of type 2 diabetes, and both NAION and type 2 DM mainly affect the elderly, it is reasonable to assume that the majority of DM patients with NAION belong to type 2 DM category [40]. However, in fact, close to one-third of diabetics are undiagnosed [41], some degree of non-differential misclassification of DM is likely to exist in some studies. All the included studies in this analysis did not specify type 1 or type 2 DM, and a small portion of DM cases were self-reported rather than clinically diagnosed. The above all may underestimate the association between DM and NAION. Therefore, it is plausible to apply our results on type 2 DM rather than type 1 DM.

Overall, substantial heterogeneity was shown among 12 included studies with an *I* ^2^ value of 45.8% (*P* = 0.036 for test of between-study heterogeneity), and majority of heterogeneity was from studies by Pinna et al [39] and Weger et al [24]. Heterogeneity was significantly decreased when these two studies were excluded (*I* ^2^ = 0.0%, p = 0.00). However, sensitivity analysis indicated that association result was very robust in the selected studies, with ORs ranging from 1.4 to 1.8. No matter which one of these studies were excluded from analysis, the association was all statistically significant. These results suggest that the association of DM with NAION is unlikely to be due to the selection bias.

Published researches suggested that ethnic differences exist in the incidence of NAION, with the highest incidence in Caucasians and the lowest in Africans [42]. Similarly, the incidence of DM varies among ethnic groups, incidence of DM in Asia was reported to be different from other parts of the world [43] and the size of the retinal arteriolar and venous calibers varies in different ethnicities as well [44]. However, our meta-regression analysis revealed no significant difference of geographical area on the association between DM and NAION. That may be partly attributed to the fact that only a small number of studies from United States, Greece, Italy, Austria, UK and Israel, and none of the studies from East Asia were included due to limited availability of published literature resources. Although the Israel is in Asia, the socioeconomic status, demographic composition of the population and lifestyle are more close to that in Europe.

In this meta-analysis, no evidence of publication bias was seen based on the funnel plot or in Begg or Egger's test (Egger *P* = 0.07 and Begg *P* = 0.13). However, it should be noted that several factors other than publication bias can affect these statistical tests. For instance, their validity and the interpretation had been debated [45], such as information bias and selection bias. The cause of DM may be the obesity and smoking. Thus, obesity and smoking may explain the increased risk of NAION in patients with DM. Whatsoever, it may be desirable to exclude all the confounding factors of NAION in our meta-analysis. However, it is impossible due to the lack or inaccessibility of data.

The underlying mechanisms of the positive correlation between DM and NAION are as yet unknown. Many studies found a reduced optic nerve head blood flow in the NAION patients [3], [46]–[48], indicating the insufficient circulation may be the pathologic factor. Hyperglycemia can promote vasostatic perfusion deficiency by multiple biochemical abnormalities such as polyol pathway, advanced glycation end products (AGEs), increased oxidative stress, activation of the protein kinase C (PKC)-β pathway and angiogenic factors, resulting in cellular damage such as the vascular endothelium and pericytes, abnormal hemodynamics and autoregulation [49], [50]. The hyperglycemia also leads to leukostasis that predisposes to capillary occlusion [49], [51] These changes in DM make the optic nerve prone to perfusion deficiency, and result in development of NAION [52] if hyperglycemia is not properly managed.

In conclusion, our meta-analysis results suggest that diabetic patients might be at increased risk of NAION. Thus, an increase of the prevalence of NAION may ensue the world-wide increase of DM. Our findings may have important clinical and public health significance, such as alerting the endocrinologists to pay attention to DM patients' visual symptoms indicative of NAION, and the neuro-ophthalmologists to concern about the blood glucose control of NAION patients presented with DM. The proper management of DM could potentially decrease the risk for NAION. The results of our study need further validation from large cohort studies or intervention trials, and future studies are needed to investigate the mechanisms underlying the association between DM and NAION if proved true.

## Supporting Information

Checklist S1 **PRISMA checklist.**
(DOC)
